# Benefit of switching to mepolizumab from omalizumab in severe eosinophilic asthma based on patient characteristics

**DOI:** 10.1186/s12931-021-01733-9

**Published:** 2021-05-10

**Authors:** Mark C. Liu, Bradley Chipps, Xavier Munoz, Gilles Devouassoux, Miguel Bergna, Steven G. Smith, Robert G. Price, Dmitry V. Galkin, Jay Azmi, Dalal Mouneimne, Frank C. Albers, Kenneth R. Chapman

**Affiliations:** 1 Divisions of Allergy and Clinical Immunology, Pulmonary and Critical Care Medicine, Johns Hopkins Asthma and Allergy Center, Baltimore, MD USA; 2 Capital Allergy and Respiratory Disease Center, Sacramento, CA USA; 3 Pulmonology Department, Hospital Universitari Vall d’Hebron, Barcelona, Spain; 4 Ciber Enfermedades Respiratorias, Madrid, Spain; 5 Service de Pneumologie, Hôpital de la Croix Rousse, Hospices Civils de Lyon, UCB Lyon, Lyon, France; 6 Respiratory Research, CEMER, Vicente Lopez, Buenos Aires, Argentina; 7 Respiratory Therapeutic Area, GSK, Research Triangle Park, NC USA; 8 Biostatistics, GSK, Stevenage, Hertfordshire UK; 9 Global Respiratory Medical Franchise, GSK, Research Triangle Park, NC USA; 10 Respiratory TAU, GSK, Uxbridge, Middlesex UK; 11 Global Medical Affairs, GSK House, Brentford, Middlesex UK; 12 Asthma and Airway Centre, University Health Network, University of Toronto, Toronto, ON Canada; 13 Present Address: Chiesi USA, Cary, NC USA; 14 Present Address: Avillion US Inc, Northbrook, IL USA

**Keywords:** Asthma, Asthma treatment, Biologics, Eosinophils

## Abstract

**Background:**

The OSMO study assessed the efficacy of switching to mepolizumab in patients with severe eosinophilic asthma that was uncontrolled whilst receiving omalizumab. The objective of this analysis was to assess the proportion of patients achieving pre-defined improvements in up to four efficacy outcomes and the relationship between patient baseline characteristics and treatment response.

**Methods:**

This was a post hoc analysis of OSMO study data (GSK ID:204471; ClinicalTrials.gov No. NCT02654145). Patients with severe eosinophilic asthma uncontrolled by high-dose inhaled corticosteroids, other controller(s) and omalizumab subcutaneously (≥ 4 months) were switched to mepolizumab 100 mg administered subcutaneously. Endpoints included the proportion of responders—i.e. patients achieving a pre-defined clinical improvement in ≥ 1 of the following outcomes: (1) Asthma Control Questionnaire (ACQ)-5 score (≥ 0.5-points), (2) St George’s Respiratory Questionnaire (SGRQ) total score (≥ 4-points), (3) pre-bronchodilator forced expiratory volume in 1s (FEV_1_; ≥ 100 mL), all at Week 32, and (4) annualised rate of clinically significant exacerbations (≥ 50% reduction).

**Results:**

Of the 145 patients included, 94%, 83%, 63% and 31% were responders for ≥ 1, ≥ 2, ≥ 3 and 4 outcomes, respectively; 75% and 78% were ACQ-5 and SGRQ score responders, and 50% and 69% were FEV_1_ and exacerbation responders. Subgroup analyses demonstrated improvements irrespective of baseline blood eosinophil count, prior omalizumab treatment regimen/duration, comorbidities, prior exacerbation history, maintenance oral corticosteroid use, ACQ-5 and SGRQ scores, and body weight/body mass index.

**Conclusions:**

After switching to mepolizumab, almost all patients with uncontrolled severe eosinophilic asthma on omalizumab achieved a beneficial response in ≥ 1 clinical outcome. Improvements were observed regardless of baseline characteristics.

*Trial registration* This manuscript is a post hoc analysis of data from the OSMO study. ClinicalTrials.gov, NCT02654145. Registered January 13, 2016.

**Supplementary Information:**

The online version contains supplementary material available at 10.1186/s12931-021-01733-9.

## Background

Severe asthma is characterised by frequent, persistent respiratory symptoms, exacerbations and reduced health-related quality of life (HRQoL), and can remain uncontrolled despite the regular use of inhaled corticosteroids (ICS) and additional controller therapies [1]. Distinct phenotypes of severe asthma have been described, including severe eosinophilic asthma and severe allergic asthma [1, 2]. However, there is often overlap in severe asthma phenotypes and as such, patients may be eligible for more than one of the currently available biologic therapies [3].

Mepolizumab is a humanised, monoclonal antibody that binds to and inactivates interleukin (IL)-5, inhibiting IL-5 signalling and blocking eosinophil survival and proliferation [4]. It is approved for the treatment of patients with severe eosinophilic asthma ≥ 6 years of age in multiple regions worldwide, for patients with eosinophilic granulomatosis with polyangiitis in several countries including the USA, Japan and Canada, and has recently been approved for use in patients with hyperesoinophilic syndrome in the US [5, 6]. Several clinical trials have shown that compared with placebo, mepolizumab reduces the rate of clinically significant exacerbations and maintenance oral corticosteroid (OCS) dose, and also improves asthma control, HRQoL and lung function in patients with severe eosinophilic asthma [7–10]. Furthermore, improvements with mepolizumab have been demonstrated in several subgroup analyses based on patient characteristics, in which characteristics such as high blood eosinophil count and comorbid upper airway diseases were associated with greater mepolizumab treatment responses [11–14]. Omalizumab is an anti‐immunoglobulin-E antibody indicated for use as an add-on treatment in patients ≥ 6 years of age with moderate‐to‐severe allergic asthma [15]. In patients with severe asthma, omalizumab treatment has been shown to decrease exacerbation rates, improve asthma control and HRQoL compared with placebo [16–18]. However, not all patients treated with omalizumab achieve adequate symptom control and reduced exacerbations [19, 20].

The OSMO study demonstrated that after directly switching from omalizumab to mepolizumab, patients with uncontrolled severe eosinophilic asthma experienced clinically significant improvements in asthma control, HRQoL, lung function and the rate of clinically significant exacerbations, with no tolerability issues reported [13]. It is of clinical interest to determine what proportion of patients respond to mepolizumab treatment, and whether patient characteristics affect this response, after switching from omalizumab. The objective of this post hoc analysis of the OSMO study was to assess the clinical benefit of a direct switch from omalizumab to mepolizumab, by performing a responder analysis of several efficacy outcomes (asthma control, HRQoL, lung function and clinically significant exacerbations). We also sought to determine the relationship between patient baseline characteristics and these efficacy outcomes.

## Methods

### Study design

This was a post hoc analysis of the multicentre, open-label, single-arm, 32-week OSMO study (GSK ID:204471; NCT02654145) [13]. Details of the OSMO study have been published previously [13]. In brief, patients with uncontrolled severe eosinophilic asthma treated with omalizumab (for at least 4 months) were switched directly (without a wash-out period) to mepolizumab 100 mg subcutaneously every 4 weeks for 32 weeks. Eligible patients had: (1) a blood eosinophil count of ≥ 150 cells/µL at screening (or ≥ 300 cells/µL in the past year); (2) ≥ 2 exacerbations in the year prior to screening (≥ 1 exacerbation during omalizumab treatment if receiving omalizumab for ≥ 8 months within the prior year); (3) Asthma Control Questionnaire (ACQ)-5 score ≥ 1.5 at both screening and baseline visits, despite receiving high-dose inhaled corticosteroids and ≥ 1 additional controller(s) [13].

This study was conducted in accordance with International Conference for Harmonization Good Clinical Practice, applicable country‐specific requirements and ethical principles outlined in the Declaration of Helsinki. All patients provided written informed consent prior to any study‐related activities. The study was approved by local ethics review boards of the participating sites.

### Endpoints and assessments

The study endpoint assessed in this analysis was the proportion of responders, i.e. patients achieving a pre-defined clinical improvement in ≥ 1 of the following outcomes, after switching from omalizumab to mepolizumab: (1) asthma control, measured by ACQ-5 score; (2) HRQoL, measured by St George’s Respiratory Questionnaire (SGRQ) total score; (3) lung function, measured by pre-bronchodilator forced expiratory volume in 1s (FEV_1_); and (4) the annualised rate of clinically significant exacerbations. A clinically significant exacerbation was defined as a worsening of asthma that required systemic corticosteroids (SCS), hospitalisation or an emergency department visit. SCS had to be delivered orally or intravenously for ≥ 3 days, or as a single intramuscular dose; for patients on maintenance SCS, the dose had to be at least twice the existing dose for ≥ 3 days. Other endpoints included the proportion of responders for each of the four individual clinical efficacy outcomes.

Clinical benefit was defined according to the established or proposed minimum clinically important differences (MCID) in treatment response for each outcome where available [21–23]. In this analysis, patients were categorised as achieving clinical benefit (responders) in each outcome if the following criteria were observed: (1) ACQ-5 score improvement from baseline of ≥ 0.5 points [22] at Week 32; (2) SGRQ total score improvement from baseline of ≥ 4 points [21] at Week 32; (3) pre-bronchodilator FEV_1_ improvement from baseline of ≥ 100 mL [23] at Week 32; and (4) a reduction of ≥ 50% in the annualised rate of clinically significant exacerbations during the study treatment period versus the previous year. Patients who prematurely discontinued mepolizumab treatment were considered non-responders within the assessment of each efficacy outcome.

### Statistical analysis

The proportion of responders across each of the four clinical efficacy outcomes was summarised using descriptive statistics. Change from baseline/prior year for each efficacy outcome was further analysed by baseline patient subgroups. Separate analysis models were used to evaluate each of the following patient subgroups: baseline blood eosinophil count (≥ 150, ≥ 300, ≥ 400, or ≥ 500 cells/µL), prior omalizumab treatment regimen (2-weekly or 4-weekly dosing), prior omalizumab treatment duration (< 1.5, ≥ 1.5– < 4, or ≥ 4 years), presence of additional comorbidities (nasal polyps [determined following physical examination by their treating physician], aspirin/non-steroidal anti-inflammatory drug [NSAID] intolerance [determined via patient medical history], or gastroesophageal reflux disease [GERD; determined via patient medical history]), exacerbations in the prior year (≤ 2, 3 or ≥ 4), requirement for maintenance OCS at baseline (use or no use), baseline ACQ-5 score quartiles (< 2.5, ≥ 2.5– < 3.0, ≥ 3.0– < 3.5 or ≥ 3.5), baseline SGRQ total score quartiles (< 45, ≥ 45– < 55, ≥ 55– < 70 or ≥ 70), body weight quartiles (< 70, 70– < 80, 80– < 95 or ≥ 95 kg) and body mass index (BMI) quartiles (< 25, 25– < 30, 30– < 35 or ≥ 35 kg/m^2^).

ACQ-5 scores, SGRQ total scores and lung function endpoints were analysed separately for each subgroup using mixed model repeated measures with covariates of region, baseline maintenance OCS use, exacerbations in the year prior to the study and visit. Exacerbations were analysed separately for each subgroup using a generalised estimating equation model assuming a negative binomial distribution with a covariate of treatment period and logarithm of time as an offset variable.

## Results

### Responder analysis

**Fig. 1:**
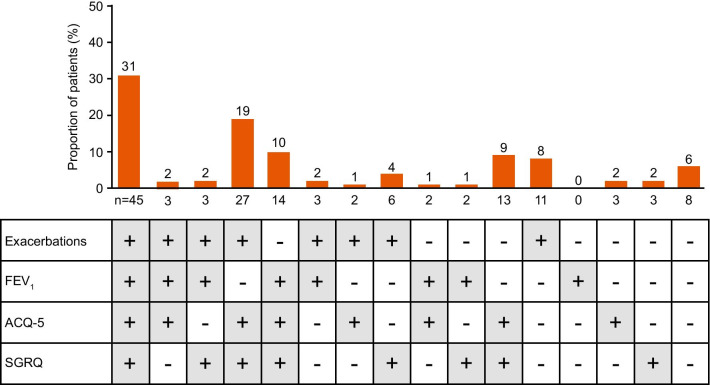
Proportion of efficacy outcome responders* for ACQ-5 score, SGRQ total score, pre-bronchodilator FEV_1_, and exacerbations. *Responders were defined as: ACQ-5 score improvement from baseline of ≥ 0.5-points at Week 32; SGRQ total score improvement from baseline of ≥ 4 points at Week 32; pre-bronchodilator FEV_1_ improvement from baseline of ≥ 100 mL at Week 32; ≥ 50% reduction in the annualised rate of clinically significant exacerbations during the study treatment period versus the previous year; patients who discontinued mepolizumab treatment but remained within the study (n = 2) were considered non-responders in this analysis. *ACQ-5* Asthma Control Questionnaire-5, *FEV*_*1*_ forced expiratory volume in 1s, *SGRQ* St George’s Respiratory Questionnaire

The intent-to-treat (ITT) population included 145 patients who switched directly from omalizumab to mepolizumab. Of these patients, 137 (94%) were identified as responders for at least one of the four efficacy outcomes, 120 (83%) were responders for at least two outcomes, 92 (63%) were responders for at least three outcomes, and 45 (31%) were responders for all four efficacy outcomes (Fig. 1). Two patients discontinued mepolizumab treatment due to adverse events (electrocardiogram QT prolonged, n = 1; urticaria, n = 1) but remained within the study and were considered non-responders in this analysis. In total, 75% and 78% of patients were ACQ-5 and SGRQ total score responders (≥ 0.5-points and ≥ 4-point improvement), respectively, at Week 32, 50% were pre-bronchodilator FEV_1_ responders (≥ 100 mL improvement) at Week 32, and 69% were clinically significant exacerbation responders (≥ 50% reduction in annualised rate) (Fig. 1).

Overall patient baseline characteristics for the OSMO study have been published previously [13]. A summary of patient baseline characteristics by responder status (number of efficacy outcomes in which a pre-defined clinical improvement was achieved) following the switch to mepolizumab from omalizumab is shown in Table 1. Patients who achieved a response in all four efficacy outcomes typically experienced a greater number of exacerbations in the prior year, had lower pre- and post-bronchodilator FEV_1,_ and had a greater occurrence of nasal polyps versus those achieving fewer pre-defined clinical improvements. Additionally, patients in the 0 benefits group (n = 8) appeared to have a longer duration of asthma, fewer comorbidities, fewer exacerbations in the prior year, and better asthma control and HRQoL (as indicated by ACQ-5 and SGRQ scores) compared with the responder subgroups.

**Table 1:** Summary of patient baseline characteristics by number of observed clinical benefits (responder analysis)

Baseline characteristics	Non-responders	Responders						
	0 clinical benefits (N = 8)	≥ 1 clinical benefit (N = 137)	≥ 2 clinical benefits (N = 120)	≥ 3 clinical benefits (N = 92)	≥ 4 clinical benefits (N = 45)	< 2 clinical benefits (N = 25)	< 3 clinical benefits (N = 53)	< 4 clinical benefits (N = 100)
Age, years, mean (SD)	49.5 (19.64)	53.8 (13.48)	54.2 (13.54)	54.3 (13.97)	53.9 (13.18)	50.5 (15.05)	52.3 (13.61)	53.5 (14.17)
Gender, female, %	75	58	58	61	73	68	57	53
Race, n (%)								
White	6 (75)	122 (89)	107 (89)	82 (89)	39 (87)	21 (84)	46 (87)	89 (89)
Asian	0	5 (4)	3 (3)	2 (2)	1 (2)	2 (8)	3 (6)	4 (4)
Black or African American	2 (25)	9 (7)	9 (8)	7 (8)	4 (9)	2 (8)	4 (8)	7 (7)
Mixed	0	1 (< 1)	1 (< 1)	1 (1)	1 (2)	0	0	0
BMI, kg/m^2^, mean (SD)	29.8 (5.78)	30.2 (6.31)	30.0 (6.15)	30.1 (6.18)	30.0 (5.70)	31.1 (6.89)	30.4 (6.47)	30.3 (6.53)
Duration of asthma, years, mean (SD)	30.9 (21.47)	25.3 (16.54)	25.0 (16.62)	24.4 (15.63)	26.2 (16.49)	28.5 (17.71)	27.7 (18.64)	25.3 (17.02)
Comorbidities at screening, n (%)								
Allergic rhinitis	1 (13)	28 (20)	23 (19)	17 (18)	11 (24)	6 (24)	12 (23)	18 (18)
Nasal polyps	0	20 (15)	20 (17)	15 (16)	10 (22)	0	5 (9)	10 (10)
Baseline maintenance OCS therapy, n (%)	0	35 (26)	31 (26)	20 (22)	8 (18)	4 (16)	15 (28)	27 (27)
Baseline maintenance OCS, mg/day, median (range)	0	10.0 (4–40)	10.0 (4–40)	10.0 (4–40)	5.0 (5–40)	7.5 (5–20)	10.0 (5–30)	10.0 (4–40)
Exacerbations in previous 12 months, mean (SD)	2.5 (1.07)	3.3 (2.71)	3.4 (2.79)	3.5 (3.10)	3.7 (3.52)	2.7 (1.74)	2.9 (1.55)	3.1 (2.14)
Pre-BD % predicted FEV_1_ at baseline, mean (SD)	52.9 (23.91)	59.9 (17.57)	59.5 (17.71)	59.4 (17.55)	57.4 (16.79)	59.5 (19.40)	59.7 (18.76)	60.5 (18.44)
Pre-BD FEV_1_ at baseline, mL, mean (SD)	1420 (550)	1780 (690)	1770 (700)	1760 (670)	1610 (570)	1690 (580)	1760 (700)	1820 (720)
Post-BD FEV_1_ at baseline, mL, mean (SD)	1590 (650)	2020 (800)	2010 (810)	2000 (770)	1840 (660)	1920 (730)	1990 (850)	2060 (850)
Baseline ACQ-5 score, mean (SD)	2.83 (0.705)	3.21 (0.947)	3.22 (0.942)	3.25 (0.881)	3.21 (0.832)	3.06 (0.923)	3.09 (1.030)	3.18 (0.985)
Baseline SGRQ total score, mean (SD)	52.7 (15.32)	56.8 (17.49)	57.2 (18.00)	56.1 (17.94)	56.8 (17.36)	53.5 (13.78)	57.5 (16.43)	56.5 (17.44)
Baseline blood eosinophil count, geometric mean (SD logs)	290 (0.884)	290 (1.151)	290 (1.206)	320 (1.220)	310 (1.403)	290 (0.722)	250 (0.959)	290 (0.998)
Prior omalizumab therapy								
Duration of prior omalizumab use, median months (range)	41.3 (6–63)	29.4 (4–161)	29.7 (4–161)	29.4 (5–161)	28.7 (5–129)	27.4 (6–81)	30.1 (4–104)	30.5 (4–161)
Frequency of prior omalizumab dosing, n (%)								
2-weekly	4 (50)	71 (52)	61 (51)	50 (54)	23 (51)	14 (56)	25 (48)	52 (53)
4-weekly	4 (50)	65 (48)	58 (49)	42 (46)	22 (49)	11 (44)	27 (52)	47 (47)
Prior omalizumab monthly dose, mg, median (range)	300 (150–900)	450 (100–1200)	450 (100–1200)	450 (100–1200)	450 (100–1200)	525 (150–1200)	450 (100–1200)	450.0 (100–1200)

Clinical benefit was defined according to the treatment response of four different efficacy outcomes: ACQ-5 score improvement from baseline of ≥ 0.5-points at Week 32, SGRQ total score improvement from baseline of 4-points at Week 32, FEV_1_ improvement from baseline of ≥ 100 mL at Week 32, a reduction of ≥ 50% in annualised exacerbation rate during the study treatment period versus the previous year

*ACQ-5* Asthma Control Questionnaire-5, *BD* bronchodilator, *BMI* body mass index, *FEV*_*1*_ forced expiratory volume in 1s, *OCS* oral corticosteroid, *SD* standard deviation, *SGRQ* St George’s Respiratory Questionnaire

### Subgroup analyses of mepolizumab response based on patient characteristics

#### Baseline blood eosinophil count

**Fig. 2:**
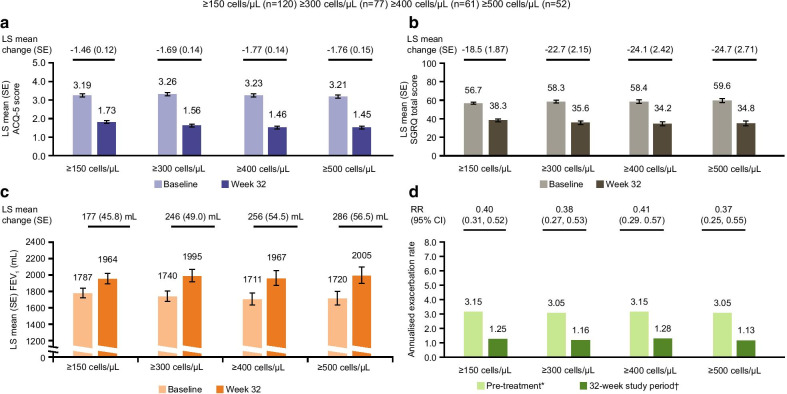
Efficacy of switching to mepolizumab from omalizumab by blood eosinophil count thresholds at baseline. *Pre-treatment refers to the 12 months prior to screening; †32-week study period refers to the time between first dose of mepolizumab and study conclusion, regardless of treatment discontinuation. Rate ratio reflecting annualised clinically significant exacerbation rate during 32-week study period compared with rate during pre-treatment period; MCID for ACQ-5 and SGRQ is 0.5 points and 4 points, respectively; error bars represent SE. *ACQ* Asthma Control Questionnaire, *CI* confidence interval, *FEV*_*1*_ forced expiratory volume in 1s, *LS* least squares, *MCID* minimum clinically important difference, *RR* rate ratio, *SE* standard error, *SGRQ* St George's Respiratory Questionnaire

A total of 120 (83%), 77 (53%), 61 (42%) and 52 (36%) patients had a baseline blood eosinophil count ≥ 150, ≥ 300, ≥ 400 and ≥ 500 cells/µL, respectively. Patients switching to mepolizumab from omalizumab demonstrated improvements in all efficacy endpoints irrespective of baseline blood eosinophil count. Improvements from baseline at Week 32 in ACQ-5 score (Fig. 2a), SGRQ total score (Fig. 2b) and pre-bronchodilator FEV_1_ (Fig. 2c) generally increased with increasing baseline blood eosinophil count. Improvements in ACQ-5 score with mepolizumab at Week 32 increased from a least squares (LS) mean change (standard error [SE]) of 1.46 (0.12) in patients with baseline blood eosinophils counts ≥ 150 cells/µL to a LS mean change (SE) of 1.76 (0.15) in patients with baseline blood eosinophils counts ≥ 500 cells/µL (Fig. 2a). The annualised rate of clinically significant exacerbations was reduced by a similar level across all baseline blood eosinophil count subgroups (60% for the ≥ 150 cells/µL subgroup, 62% for ≥ 300 cells/µL, 59% for ≥ 400 cells/µL, and 63% for ≥ 500 cells/µL) (Fig. 2d).

### Prior omalizumab treatment regimen and duration

Of the 145 patients in the ITT population, 144 were included in the subgroup analyses by prior omalizumab treatment. One patient who had previously received omalizumab at a non-approved regimen of every 3 weeks was excluded.

**Fig. 3:**
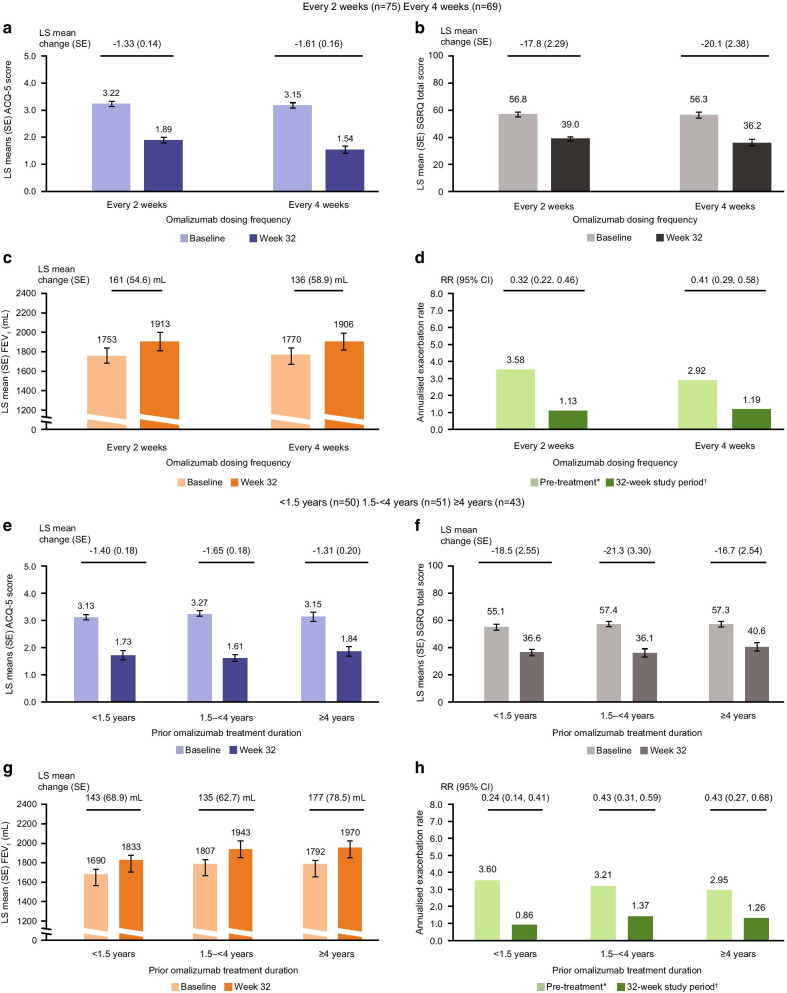
Efficacy of switching to mepolizumab from omalizumab by prior treatment regimen and omalizumab treatment duration. *Pre-treatment refers to the 12 months prior to screening; †32-week study period refers to the time between first dose of mepolizumab and study conclusion, regardless of treatment discontinuation. Rate ratio reflecting annualised clinically significant exacerbation rate during 32-week study period compared with rate during pre-treatment period. MCID for ACQ-5 and SGRQ is 0.5 points and 4 points, respectively; error bars represent SE. *ACQ* Asthma Control Questionnaire, *CI* confidence interval, *FEV*_*1*_ forced expiratory volume in 1s, *LS* least squares, *MCID* minimum clinically important difference, *RR* rate ratio, *SE* standard error, *SGRQ* St. George's Respiratory Questionnaire

In total, 75 (52%) and 69 (48%) patients had previously received omalizumab every 2 and 4 weeks, respectively. When switched to mepolizumab, mean improvements from baseline in ACQ-5 scores (Fig. 3a), SGRQ total score (Fig. 3b) and pre-bronchodilator FEV_1_ (Fig. 3c) were similar at Week 32 regardless of 2-weekly or 4-weekly prior omalizumab treatment regimen; improvements from baseline were greater than the MCID for ACQ-5 and SGRQ and above a 100 mL increase for pre-bronchodilator FEV_1_ in both regimen subgroups. Following the switch to mepolizumab, reductions in the annualised rate of clinically significant exacerbations compared with the prior 12 months were similar between patients who had previously received 2-weekly omalizumab (68% reduction) and those who had received the 4-weekly regimen (59% reduction) (Fig. 3d).

In total, 50 (35%) patients received omalizumab for < 1.5 years, 51 (35%) for 1.5– < 4 years, and 43 (30%) for ≥ 4 years. Consistent with the subgroup analysis by prior omalizumab treatment regimen, improvements in all efficacy outcomes when switching from omalizumab to mepolizumab were similar and above the MCID at Week 32 for ACQ-5 score (Fig. 3e), SGRQ total score (Fig. 3f), and above a 100 mL increase for pre-bronchodilator FEV_1_ (Fig. 3g). Following the switch to mepolizumab, reductions of 57–76% in the annualised rate of clinically significant exacerbations compared with the prior 12 months were seen across subgroups of previous omalizumab treatment duration (Fig. 3h).

### Comorbidities

**Fig. 4:**
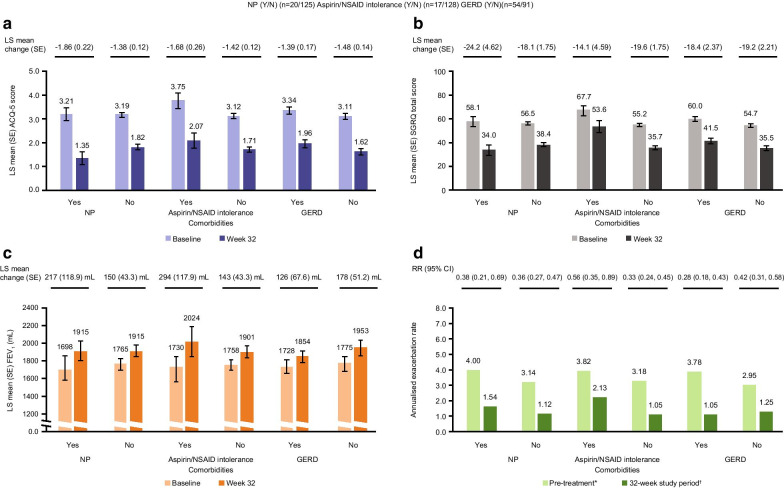
Efficacy of switching to mepolizumab from omalizumab by the presence or absence of comorbidities. *Pre-treatment refers to the 12 months prior to screening; †32-week study period refers to the time between first dose of mepolizumab and study conclusion, regardless of treatment discontinuation. Rate ratio reflecting annualised clinically significant exacerbation rate during 32-week study period compared with rate during pre-treatment period. MCID for ACQ-5 and SGRQ is 0.5 points and 4 points, respectively; error bars represent SE. *ACQ* Asthma Control Questionnaire, *CI* confidence interval, *FEV*_*1*_ forced expiratory volume in 1s, *GERD* gastroesophageal reflux disease, *LS* least squares, *MCID* minimum clinically important difference, *NP* nasal polyps, *NSAID* aspirin/non-steroidal anti-inflammatory drug, *RR* rate ratio, *SE* standard error, *SGRQ* St George's Respiratory Questionnaire

At the study screening visit, 20 (14%) patients had nasal polyps, 17 (12%) had aspirin/NSAID intolerance and 54 (37%) had GERD. Following the switch to mepolizumab from omalizumab, there was an improvement from baseline in ACQ-5 scores (Fig. 4a), SGRQ total scores (Fig. 4b) and pre-bronchodilator FEV_1_ (Fig. 4c) regardless of presence or absence of the aforementioned comorbid conditions; all improvements exceeded the MCID for ACQ-5 and SGRQ scores or a 100 mL increase for pre-bronchodilator FEV_1_ at Week 32. In patients with comorbid nasal polyps, improvements in ACQ-5 and SGRQ scores and FEV_1_ were numerically higher compared with those without nasal polyps. Switching to mepolizumab from omalizumab resulted in reductions in the annualised rate of clinically significant exacerbations of approximately 50% or greater across all comorbidity subgroups compared with the prior 12 months (Fig. 4d).

### Previous exacerbations and maintenance OCS use

**Fig. 5:**
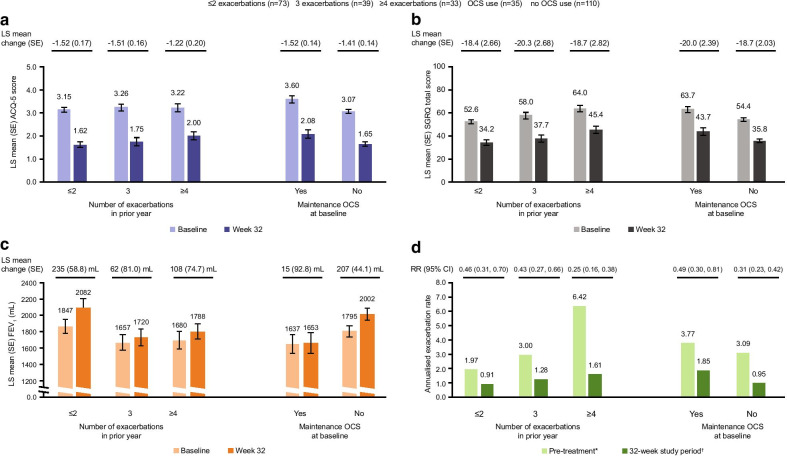
Efficacy of switching to mepolizumab from omalizumab by exacerbation history and maintenance OCS use. *Pre-treatment refers to the 12 months prior to screening; †32-week study period refers to the time between first dose of mepolizumab and study conclusion, regardless of treatment discontinuation. Rate ratio reflecting annualised clinically significant exacerbation rate during 32-week study period compared with rate during pre-treatment period. MCID for ACQ-5 and SGRQ is 0.5 points and 4 points, respectively; error bars represent SE. *ACQ* Asthma Control Questionnaire, *CI* confidence interval, *FEV*_*1*_ forced expiratory volume in 1s, *LS* least squares, *MCID* minimum clinically important difference, *OCS* oral corticosteroid, *RR* rate ratio, *SE* standard error, *SGRQ* St George's Respiratory Questionnaire

Overall, 73 (50%), 39 (27%), and 33 (23%) patients reported ≤ 2, 3 and ≥ 4 exacerbations, respectively, in the year prior to switching to mepolizumab from omalizumab. Additionally, 35 (24%) were using maintenance OCS at baseline. Regardless of prior exacerbation history and maintenance OCS use, following switch to mepolizumab from omalizumab mean changes from baseline in ACQ-5 score and SGRQ total score at Week 32 exceeded the MCID (Fig. 5a, b). Improvements from baseline in pre-bronchodilator FEV_1_ were also observed at Week 32, regardless of prior exacerbation history; numerically greater and clinically important improvements were observed in patients with fewer (≤ 2) exacerbations in the previous year (Fig. 5c). Numerically greater and clinically important improvements in FEV_1_ were observed for patients not using maintenance OCS at baseline, compared with those who were using maintenance OCS. Reductions in clinically significant exacerbations were observed in all prior exacerbation history and maintenance OCS use subgroups following switch to mepolizumab, with a trend for greater reductions in patients with more exacerbations in the prior year and in patients not using maintenance OCS at baseline (Fig. 5d).

### ACQ-5/SGRQ quartiles

A total of 35 (24%), 20 (14%), 37 (26%) and 53 (37%) patients reported baseline ACQ-5 scores of < 2.5, 2.5–< 3.0, 3.0– < 3.5 and ≥ 3.5, respectively. Additionally, 40 (28%), 31 (21%), 40 (28%) and 34 (23%) patients reported baseline SGRQ total scores of < 45, 45– < 55, 55– < 70 and ≥ 70, respectively. At Week 32 following switching to mepolizumab from omalizumab, mean improvements from baseline in ACQ-5 score and SGRQ total scores exceeded the MCID regardless of baseline ACQ-5 or SGRQ score quartiles, with the exception of asthma control in the < 2.5 baseline ACQ-5 category where the mean change was an improvement of 0.44 points (Additional file 1: Figure S1A, B); improvements from baseline were generally numerically greater in patients with higher (worse) baseline ACQ-5 scores or SGRQ total scores. Consistent improvements from baseline in pre-bronchodilator FEV_1_ and reductions in the rate of clinically significant exacerbations versus the prior year were observed regardless of baseline ACQ-5 or SGRQ score quartiles, with the exception of FEV_1_ in the < 2.5 baseline ACQ-5 category (Additional file 1: Figure S1C and D).

### Body weight/BMI quartiles

A total of 32 (22%), 32 (22%), 46 (32%) and 35 (24%) patients had a body weight at baseline of < 70, 70– < 80, 80– < 95, and ≥ 95 kg, respectively. In addition, 31 (21%), 47 (32%), 38 (26%) and 29 (20%) patients had a BMI at baseline of < 25, 25– < 30, 30– < 35, and ≥ 35 kg/m^2^, respectively. Improvements from baseline in ACQ-5 score and SGRQ total score at Week 32 exceeded the MCID for all body weight and BMI quartiles (Additional file 1: Figure S2A and B). Improvements from baseline in pre-bronchodilator FEV_1_ were observed with mepolizumab in all body weight subgroups ≥ 70 kg and all BMI subgroups ≥ 25 kg/m^2^ (Additional file 1: Figure S2C). Reductions in the rate of clinically significant exacerbations versus the prior year were observed for all body weight and BMI quartiles (Additional file 1: Figure S2D).

## Discussion

In this post hoc responder analysis of data from the OSMO study, a switch in biologic therapy to mepolizumab from omalizumab enabled almost all patients (94%) with severe eosinophilic asthma to achieve a clinical improvement in at least one of the four pre-defined efficacy outcomes of asthma control, HRQoL, lung function and clinically significant exacerbation rate. Furthermore, nearly one-third of patients in this study were responders across all four clinical efficacy outcomes. When changes from baseline/the prior year in outcomes were further analysed based on patient clinical characteristics, all subgroups generally demonstrated improvements in each of the four efficacy outcomes when switched to mepolizumab from omalizumab. A small number of patient subgroups, including patients with higher baseline blood eosinophil counts or comorbid nasal polyps, demonstrated numerically greater improvements in these efficacy outcomes than for patients with lower baseline blood eosinophil counts or without nasal polyps, suggesting greater mepolizumab treatment benefits in those groups of patients.

Although the number of patients in some subgroups was small, improvements in all four efficacy outcomes (ACQ-5 score, SGRQ total score, pre-bronchodilator FEV_1_ and rate of clinically significant exacerbations) across subgroups were generally consistent with that of the overall ITT population reported within the primary analysis of the OSMO study [13]. In addition, regardless of baseline blood eosinophil counts, prior omalizumab treatment regimen or duration, comorbidities, prior exacerbation history, maintenance OCS use, baseline ACQ-5 and SGRQ total scores, and body weight and BMI, mean improvements from baseline exceeding the MCID for ACQ-5 score and SGRQ total score were observed in all but one subgroup (ACQ score < 2.5 at baseline) [21, 22]. Clinically significant improvements in pre-bronchodilator FEV_1_ and the annual rate of clinically significant exacerbations were also observed for most subgroups. This suggests that patients uncontrolled on omalizumab switching to mepolizumab are likely to achieve clinically important improvements regardless of baseline characteristics.

Several subgroups demonstrated numerically greater mepolizumab treatment effects for certain efficacy outcomes following the switch from omalizumab; however, this pattern was not consistently observed across all efficacy outcomes studied. Two subgroups, patients with comorbid nasal polyps and those with a higher baseline blood eosinophil counts, appeared to experience greater benefits from mepolizumab treatment compared with patients without nasal polyps or lower baseline blood eosinophil counts across at least three efficacy outcomes (ACQ-5 score, SGRQ total score and pre-bronchodilator FEV_1_), in addition to reductions of at least 60% in the annualised rate of clinically significant exacerbations. Both nasal polyps and a high blood eosinophil count represent markers of increased disease severity and burden for patients with severe eosinophilic asthma, [24–26], and these results are consistent with those observed in previous studies [12–14]. Since the presence of nasal polyps and elevated peripheral blood eosinophils have both been associated with increased levels of IL-5 and elevated T helper type-2 pathway activity [27–29], we hypothesise that the mepolizumab treatment effect was most notable in patients with these characteristics as a result of its specific binding to (and therefore inactivation of) IL-5 [30]. Indeed, several previous studies in patients with severe eosinophilic asthma have shown greater mepolizumab treatment responses with increasing baseline blood eosinophil count [12, 13]. A meta-analysis of four randomised clinical trials of mepolizumab in patients with severe eosinophilic asthma also found that treatment responses to mepolizumab were numerically greater in patients with comorbid upper airway disease versus those without comorbid disease [14]. Consistent with this, the anti-IL-5 receptor monoclonal antibody benralizumab demonstrated greater treatment effects in patients with severe asthma and comorbid nasal polyps [31]. Finally, the recent Phase III SYNAPSE study demonstrated that mepolizumab reduces symptoms and need for surgery in patients with chronic rhinosinusitis with nasal polyps (unpublished data) [32].

It should be acknowledged that the results of this analysis may, in places, represent a regression toward the mean. For example, at the time of enrolment, patients may have entered the study with a randomly poor value for one or more of the assessed characteristics (ACQ-5 score, SGRQ score, FEV_1_, or exacerbation rate), which may have returned to the natural average for that patient following the initiation of treatment. This phenomenon may be reflected by the greater reductions in exacerbation rates in patients with a greater number of exacerbations in the prior year versus those with a lower number. Or, similarly, with larger improvements observed in ACQ-5 and SGRQ total scores in patients with greater ACQ-5 and SGRQ scores at baseline, respectively, versus those with lower baseline scores. Alternatively, higher baseline values in these efficacy outcomes may have provided greater potential for improvements with mepolizumab treatment.

The limitations of the OSMO study have been previously documented [13]. These included the use of a single-arm, open-label study design; that endpoints were assessed only up to 32 weeks rather than 12 months; and that the initial indications for prescribing omalizumab were not known for all patients. In addition, there was no wash-out period from omalizumab; as such the first dose of mepolizumab was administered when omalizumab had not been fully eliminated from the body. This may have contributed to the efficacy results during the omalizumab wash-out period at the beginning of the study. However, as these patients were previously uncontrolled on omalizumab, it is unlikely omalizumab contributed to observed patient treatment responses to mepolizumab during the rest of the OSMO study period. Moreover, there was no evidence of greater efficacy during the first half versus the second half of the mepolizumab treatment period, suggesting that there was no positive interaction during the potential washout period from omalizumab. The current results should also be considered in the context of the limitations of post hoc analyses. These were non-pre-specified analyses, and as such, these post hoc findings were not the primary objective of the original study. Furthermore, patient numbers were small for many of the subgroups assessed; as a result the analyses may not have been sufficiently powered to conclusively determine the influence of all the baseline characteristics investigated. These results should therefore be considered as hypothesis-generating and further studies are required to confirm our findings.

## Conclusions

In summary, this analysis demonstrates that patients with severe eosinophilic asthma who are uncontrolled on omalizumab can be effectively switched to mepolizumab to achieve clinically important improvement in efficacy outcomes, irrespective of the patient baseline characteristics studied. The analysis also suggests that patients with higher baseline blood eosinophil counts or comorbid nasal polyps may benefit the most from a direct switch to mepolizumab from omalizumab.

## Supplementary Information

**Additional file 1: Figure S1.** Efficacy of switching to mepolizumab from omalizumab by baseline ACQ-5 or SGRQ score quartiles. **Figure S2.** Efficacy of switching to mepolizumab from omalizumab by baseline body weight or BMI quartiles.

## Data Availability

Anonymised individual participant data and study documents for the parent study can be requested for further research from www.clinicalstudydatarequest.com.

## Abbreviations

ACQ: Asthma Control Questionnaire
BMI: Body mass index
FEV_1_: Forced expiratory volume in 1s
GERD: Gastroesophageal reflux disease
HRQoL: Health-related quality of life
ICS: Inhaled corticosteroids
IL: Interleukin
ITT: Intent to treat
LS: Least squares
MCID: Minimum clinically important differences
NSAID: Non-steroidal anti-inflammatory drug
OCS: Oral corticosteroid
SGRQ: St George’s Respiratory Questionnaire
SCS: Systemic corticosteroids
SE: Standard error
