# Supplementary material for: Benefit of switching to mepolizumab from omalizumab in severe eosinophilic asthma based on patient characteristics
Source: Respir Res. 2021 May 10;22:144. doi: 10.1186/s12931-021-01733-9 (PMC8111733; doi:10.1186/s12931-021-01733-9)
Supplement: Supplementary file 1 — Additional file 1: Figure S1. Efficacy of switching to mepolizumab from omalizumab by baseline ACQ-5 or SGRQ score quartiles. Figure S2. Efficacy of switching to mepolizumab from omalizumab by baseline body weight or BMI quartiles. [file 12931_2021_1733_MOESM1_ESM.docx]

## Additional File 1

**Benefit of switching to mepolizumab from omalizumab in severe eosinophilic asthma based on patient characteristics**

Mark C Liu, Bradley Chipps, Xavier Munoz, Gilles Devouassoux, Miguel Bergna, Steven G Smith, Robert G Price, Dmitry V Galkin, Jay Azmi, Dalal Mouneimne, Frank C Albers, Kenneth R Chapman

**List of figures**

**Additional Figure S1.** Efficacy of switching to mepolizumab from omalizumab by baseline ACQ-5 or SGRQ score quartiles.

**Additional Figure S2.** Efficacy of switching to mepolizumab from omalizumab by baseline body weight or BMI quartiles.

**Additional Figure S1.** Efficacy of switching to mepolizumab from omalizumab by baseline ACQ-5 or SGRQ score quartiles.


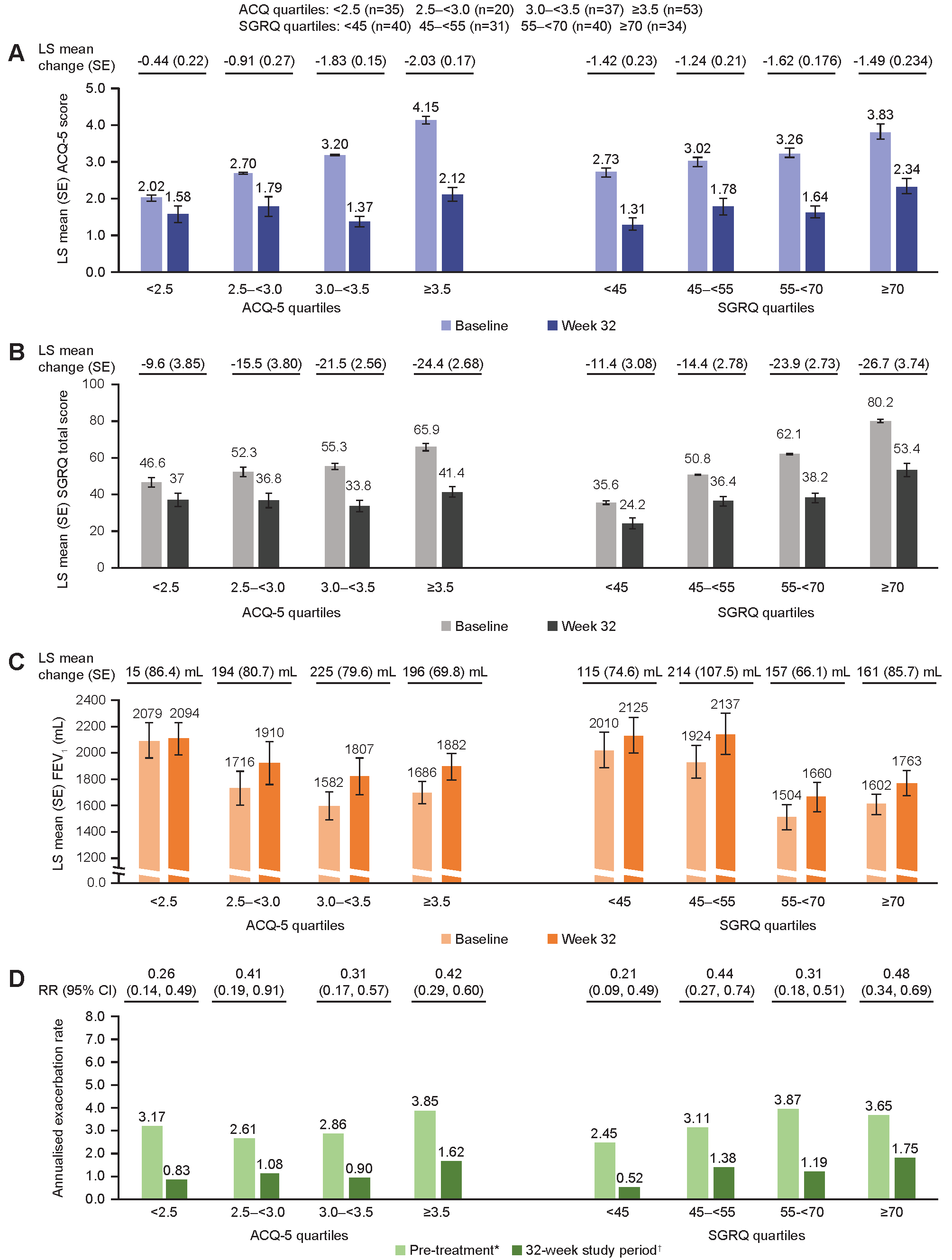


*Pre-treatment refers to the 12 months prior to screening; †32-week study period refers to the time between first dose of mepolizumab and study conclusion, regardless of treatment discontinuation. Rate ratio reflecting annualised clinically significant exacerbation rate during 32-week study period compared with rate during pre-treatment period. MCID for ACQ-5 and SGRQ is 0.5 points and 4 points, respectively; error bars represent SE.

ACQ, Asthma Control Questionnaire; CI, confidence interval; FEV_1_, Forced expiratory volume in 1 second; LS, least squares; MCID, minimum clinically important difference; RR, rate ratio; SE, standard error; SGRQ, St George's Respiratory Questionnaire.

**Additional Figure S2.** Efficacy of switching to mepolizumab from omalizumab by baseline body weight or BMI quartiles.


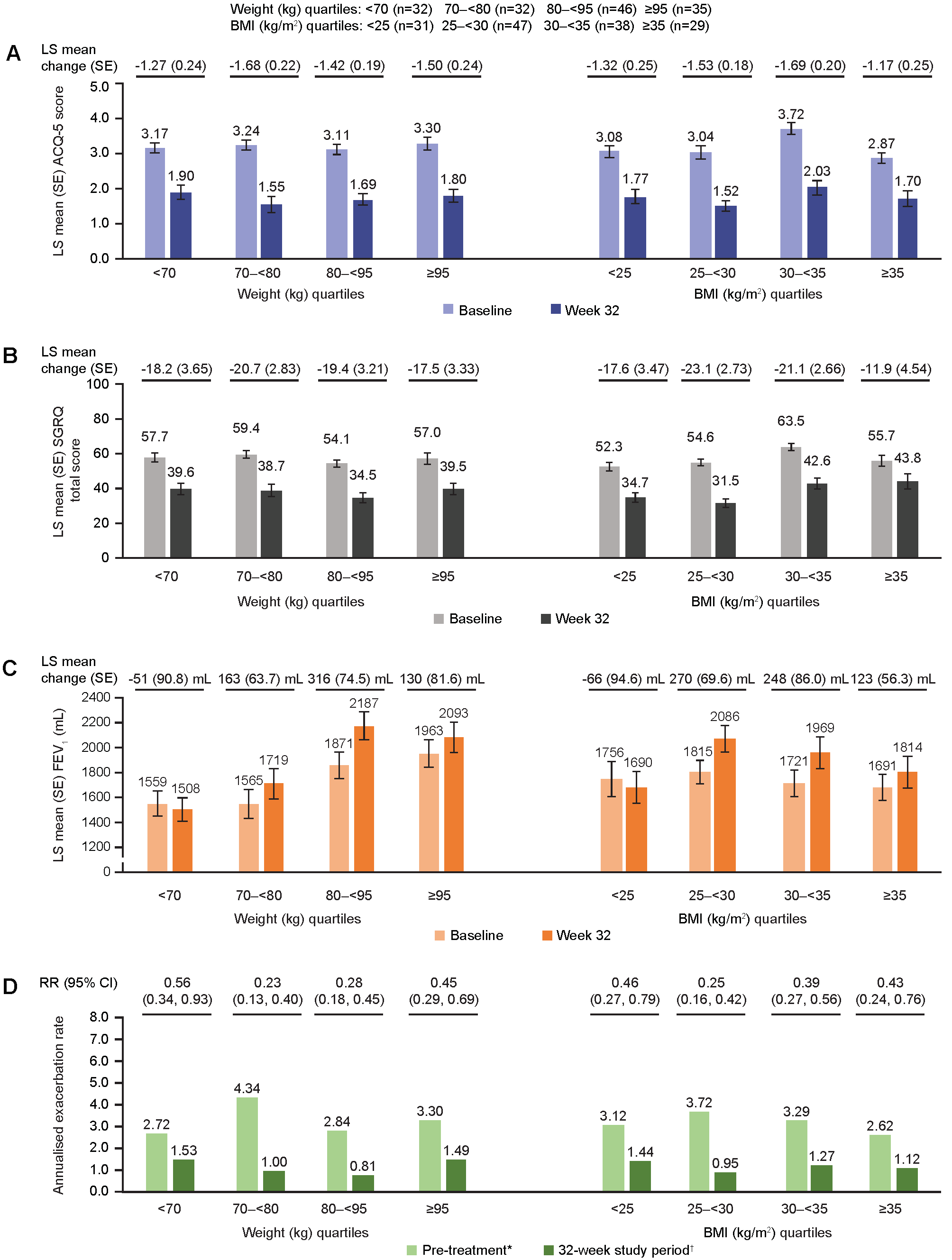


*Pre-treatment refers to the 12 months prior to screening; †32-week study period refers to the time between first dose of mepolizumab and study conclusion, regardless of treatment discontinuation. Rate ratio reflecting annualised clinically significant exacerbation rate during 32-week study period compared with rate during pre-treatment period. MCID for ACQ-5 and SGRQ is 0.5 points and 4 points, respectively; error bars represent SE.

ACQ, Asthma Control Questionnaire; BMI, body mass index; CI, confidence interval; FEV_1_, Forced expiratory volume in 1s; LS, least squares; MCID, minimum clinically important difference; RR, rate ratio; SE, standard error; SGRQ, St George's Respiratory Questionnaire.
